# Fabrication Methods and Chronic In Vivo Validation of Mechanically Adaptive Microfluidic Intracortical Devices

**DOI:** 10.3390/mi14051015

**Published:** 2023-05-09

**Authors:** Youjoung Kim, Natalie N. Mueller, William E. Schwartzman, Danielle Sarno, Reagan Wynder, George F. Hoeferlin, Kaela Gisser, Jeffrey R. Capadona, Allison Hess-Dunning

**Affiliations:** 1 Department of Biomedical Engineering, Case Western Reserve University, Cleveland, OH 44106, USA; yjk14@case.edu (Y.K.);; 2 Advanced Platform Technology Center, Louis Stokes Cleveland VA Medical Center, Cleveland, OH 44106, USA

**Keywords:** microfluidic, polymer, mechanically adaptive, neural interface, microfabrication, drug delivery

## Abstract

Intracortical neural probes are both a powerful tool in basic neuroscience studies of brain function and a critical component of brain computer interfaces (BCIs) designed to restore function to paralyzed patients. Intracortical neural probes can be used both to detect neural activity at single unit resolution and to stimulate small populations of neurons with high resolution. Unfortunately, intracortical neural probes tend to fail at chronic timepoints in large part due to the neuroinflammatory response that follows implantation and persistent dwelling in the cortex. Many promising approaches are under development to circumvent the inflammatory response, including the development of less inflammatory materials/device designs and the delivery of antioxidant or anti-inflammatory therapies. Here, we report on our recent efforts to integrate the neuroprotective effects of both a dynamically softening polymer substrate designed to minimize tissue strain and localized drug delivery at the intracortical neural probe/tissue interface through the incorporation of microfluidic channels within the probe. The fabrication process and device design were both optimized with respect to the resulting device mechanical properties, stability, and microfluidic functionality. The optimized devices were successfully able to deliver an antioxidant solution throughout a six-week in vivo rat study. Histological data indicated that a multi-outlet design was most effective at reducing markers of inflammation. The ability to reduce inflammation through a combined approach of drug delivery and soft materials as a platform technology allows future studies to explore additional therapeutics to further enhance intracortical neural probes performance and longevity for clinical applications.

## 1. Introduction

Properly functioning intracortical neural probes are crucial for their inclusion in approaches to improve patient quality of life for amputees and individuals with tetraplegia resulting from injury or disease [1,2,3,4,5,6,7]. Intracortical microelectrodes (IMEs) are designed to facilitate the detection of densely encoded neuronal activity with single-cell resolution by placing recording microelectrodes within close proximity (<100 µm) to the targeted neurons [8]. The detected signals can be translated to control of implanted functional electrical stimulation systems or external devices [4,6,7,9]. Despite the broad potential for IMEs as part of a brain–machine interface (BMI) system, clinical translation and adoption are hampered by the decline in signal quality and the loss of the number of functional recording channels due to a combination of biological and material failures driven by neuroinflammation [10].

The act of implantation and the chronic residence of recording electrodes in brain tissue activates a cell-driven neuroinflammatory response, prompting (among other things) an increased production of reactive oxygen species (ROS). ROS contribute to both biological and material failure mechanisms by damaging not only cells in the direct vicinity of the implant, but the implant substrate itself [11,12,13]. Biological failures are broadly characterized by glial scar build-up and death of nearby neurons at the implant–tissue interface, resulting in a low signal-to-noise ratio (SNR) in neural recordings [14,15]. In material failure, the microenvironment of the tissue results in oxidative damage to the material components of the intracortical neural probes [14]. Multiple studies have reported that systemic drug delivery employed to reduce inflammation has led to improved recording performance and longevity [16,17,18,19,20]. However, systemic drug delivery methods can introduce difficulties in bioavailability because of the blood–brain barrier (BBB) and low solubility [21,22,23,24,25].

Previous studies in our laboratories have demonstrated that both highly compliant neural probe structures and local anti-oxidant delivery attenuate the inflammatory responses to the implant [26,27,28,29,30,31,32]. In that initial work, a mechanically adaptive polymer nanocomposite (NC) served as the compliant implant structural material [26,27,28,29,30,31,32]. When dry, cellulose nanocrystals within the soft poly(vinyl acetate) matrix form a percolating network to produce a material that is sufficiently rigid to insert into brain tissue without buckling. Within minutes of implantation, the material softens by 2–3 orders of magnitude after implantation [27,30]. Decreased differential strain at the NC device–tissue interface [33,34,35] yielded decreased activation of both glial cells and macrophage/microglia, resulting in increased neural density near the device compared to surface-matched rigid controls [28]. Despite a larger size, the NC device demonstrated decreased tissue response compared to the smaller silicon probes.

Separately, systemic or ventricular delivery of the natural anti-oxidant resveratrol yielded improved neural density and decreased oxidative stress, indicating decreased neural inflammation [16,36]. Resveratrol-loaded NC implants released over several days post-implant resulted in decreased inflammation compared to non-releasing controls at 2 weeks. However, no significant difference was observed for the chronic 16-week time point, suggesting that sustained improvement in device–tissue integration requires sustained anti-oxidant release [29].

Direct delivery to the brain matter at the site of microelectrode implantation bypasses the BBB and allows efficient and consistent delivery of the required doses for therapeutic effect. Rigid silicon microelectrode devices with fused capillary catheters or integrated microfluidic channels have demonstrated combined intracortical recording and local drug release during intraoperative studies [37,38,39,40]. To better match the mechanical properties of the brain, microfluidic neural probes have been fabricated using many different polymers, including parylene, SU-8, and polydimethylsiloxane (PDMS) [41,42,43,44,45]. Despite an abundance of microfluidic neural probe materials and architectures, there are very few reports of such probes being used for drug delivery studies beyond intraoperative experiments at acute time points. Jeong et al. reported on programmable fluidic delivery of viruses through PDMS/polycarbonate microfluidic probe in awake, behaving animals [46]. Liu et al. reported on a 4-week study with a hybrid silicon-parylene neural probe with microfluidic delivery of minocycline [47]. The duration of release is unclear in both studies.

Here, we report on the development of a NC-based neural probe for sustained resveratrol delivery. We explored three methods of device fabrication consisting of two traditional methods used in the literature for microdevice fabrication, and one hybrid method [48,49]. The first two methods are replica micro-molding (referred to as “mold-only” in this paper for simplicity) and hot micro-embossing (“embossed-only”). Upon determining the most effective fabrication method, three probe designs were evaluated for chronic functionality and performance through an in vivo rodent study followed by post-mortem histology.

## 2. Materials and Methods

### 2.1. Microfluidic Film Fabrication

**Figure 1:**
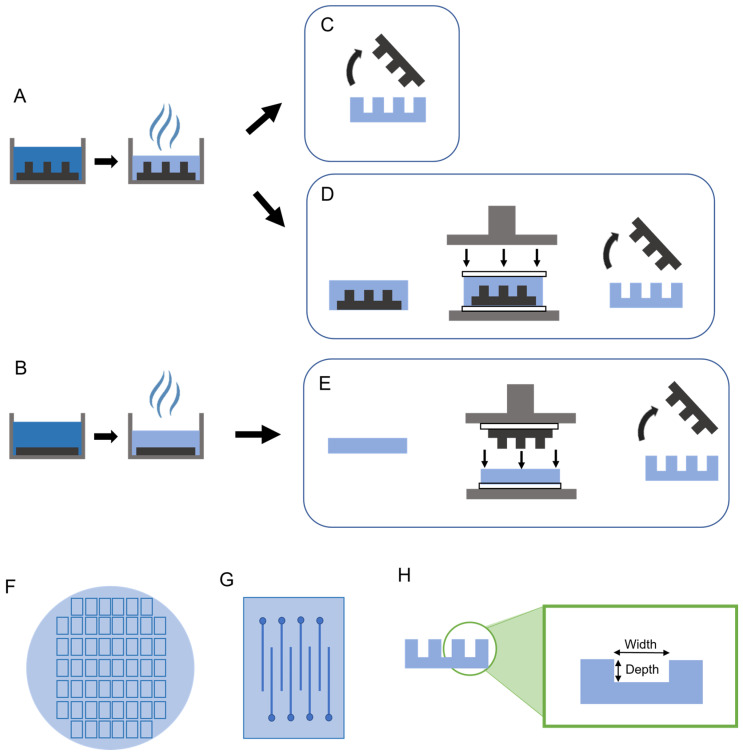
Steps for film fabrication using three methods: mold-only, emboss-only, and mold-emboss. (**A**) Solution of PVAc and tCNC in DMF is poured into a negative PDMS mold, and the solvent is allowed to evaporate in an oven to create channel layers for the mold-only and mold-emboss methods. (**B**) Same solution as in A is poured over a flat PDMS slab and the solvent evaporates in an oven to form a flat film using the emboss-only method. (**C**) The mold-only method removes the PDMS mold to form channel layer films. (**D**) The mold-emboss method takes the film and mold prior to separation and embosses between two sheets of Teflon shown here as white rectangles. (**E**) The emboss-only method removes the film from the flat PDMS slab and embosses the PDMS mold over the film between Teflon sheets. In both the mold-emboss and emboss-only methods, the PDMS molds are removed after embossing. (**F**) Schematic representation of a 100 mm-diameter film, with rectangular chips. (**G**) Each chip consists of 16 channels, corresponding to 16 probes (8 channels shown in the schematic). (**H**) The channel layer film with channels has the width defined as the wall-to-wall distance of the channel. The depth is defined as the top of the film surface to the bottom of the channel trough distance.

The process of fabricating NC-based microfluidic neural probe channel layers is shown in Figure 1. Each step is detailed in the following sections.

#### 2.1.1. PDMS Mold Fabrication

An SU-8 2015 (MicroChem Corp., Westborough, MA, USA) channel pattern was photolithographically defined on a 100 mm-diameter silicon wafer to serve as a positive mold for a polydimethylsiloxane (PDMS) negative mold. Sylgard 184 elastomer was mixed in a 10:1 ratio and degassed in a desiccator chamber connected to a vacuum for 30 min while the SU-8 mold was placed in a PTFE dish. After degassing, 330 g of PDMS prepolymer solution was carefully poured over the mold and cured in the oven at 60 °C for 1 h, resulting in a 3 mm-thick mold. The oven was then turned off and the mold was allowed to cool slowly overnight. Once the PDMS mold was completely cool, it was removed from both the PTFE dish and the SU-8 mold, for use in subsequent steps.

#### 2.1.2. Film Fabrication

The same nanocomposite (NC) composition was used for all films and followed established protocols (Figure 1) [26,50,51]. Tunicate cellulose nano-crystals (tCNCs) (Tunicate Cellulose Nanocrystals, sulfuric acid hydrolysis, Cellulose Lab, Fredericton, NB, Canada) were dispersed in dimethyl formamide (DMF) (Fisher Scientific, Hampton, NH, USA) by sonicating for 6 h. Separately, polyvinyl acetate (PVAc, Average Mw ~100,000, (189480, Sigma-Aldrich, St. Louis, MO, USA) was dissolved in DMF with the aid of magnetic stirring for 6 h. Once even dispersion of tCNCs in DMF was confirmed by demonstrating birefringence through cross-polarizing films, the tCNC + DMF solution was added to the PVAc + DMF solution to result in a 15% *w*/*w* film of tCNCs within the PVAc [26]. After mixing, the solutions were poured into a Teflon dish containing either a planar PDMS slab or a negative PDMS mold of the microfluidic channel pattern (Figure 1A,B). The films were dried by evaporating the DMF in a vacuum oven at 60 °C for one week.

After DMF evaporation, mold-only films were carefully separated from the PDMS mold using forceps (Figure 1C). To prevent brittle film fracture during the release step, droplets of DI water were used to soften the NC film during the separation step. Subsequently, the film was returned to the vacuum oven for drying under vacuum for 30 min at 60 °C.

Three methods were used to form microfluidic channels within NC. Emboss-only films were cast on planar PDMS slabs, then separated from the PDMS slab with the aid of forceps and water-induced softening (Figure 1B). The film was then placed under a PDMS channel-layer mold in between polyterafluoroethylene (PTFE) sheets, and embossed for 1 h at 90 °C in a heat press (Figure 1E). The hybrid method implemented parts of both the mold-only and emboss-only methods (Figure 1D). Prior to removing the mold-embossed films from the PDMS mold, the dry film was first pressed into the PDMS mold for 1 h at 90 °C with a heat press.

Cover layers were created similar to emboss-only films, where the NC solution was cast into a Teflon dish with a flat PDMS slab. For mold-only films, the cover layers were then taken off the PDMS slabs carefully with forceps. All cover layer films, regardless of use for mold-only or mold-emboss channel layer film were first pressed before removing from the PDMS slabs.

The resulting films of all methods were 100 mm in diameter and consisted of 84 rectangular chips (schematic representation shown in Figure 1F) with 16 channels per chip (schematic representation shown in Figure 1G). The films were separated into individual chips by razor-cutting, for subsequent chip-scale fabrication steps. On the cross-section (Figure 1H), the channels can be viewed clearly.

### 2.2. Film Characterization

Microfluidic channel layer films with 100 µm-wide channels were analyzed using stylus profilometry and high-magnification optical microscopy (Keyence, VHX-7000, Osaka, Japan) to determine channel depth (*n* = 6 per group) and width (*n* = 15 per group) dimensions (Figure 1H) and fidelity to the PDMS molds.

Tensile testing (Instron 5965 Dual Column, Instron, Norwood, MA) of ASTM D1708 standard test specimens was used to assess the effects of compression on the mechanical properties of the films. Samples were laser-micromachined from as-cast or pressed planar films to correspond to the fabrication conditions used for the mold-only and mold-embossed channel layer fabrication methods, respectively. The lateral dimensions of each sample were confirmed by measuring them under a microscope before testing. The thickness of each sample was measured using a micrometer. Samples were tested using a clamp force of 70 psi and a tensile strain rate of 10%/min. Each group had at least three replicates.

### 2.3. Device Fabrication

#### 2.3.1. Fabrication of Microfluidic Probes

**Figure 2:**
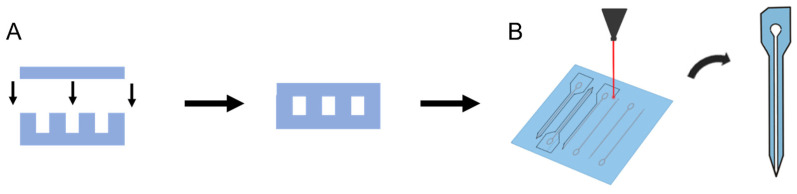
(**A**) Cover layers and channel layers were aligned and thermally bonded. (**B**) Afterward, individual microfluidic probes were micromachined and removed from the bonded chips.

Cover layers with inlet holes were laser-micromachined from planar NC films. Channel and cover layer film chips approximately 7 mm × 9 mm in size were soaked overnight in DI water at 20 °C to plasticize the films. The water-saturated films were aligned and thermally bonded on a hotplate at 50 °C for 15 min (Figure 2A). Each bonded microfluidic probe chip had features corresponding to 16 microfluidic devices, which were laser-micromachined to yield individual microfluidic neural probes (Figure 2B).

#### 2.3.2. Probe Design

**Figure 3:**
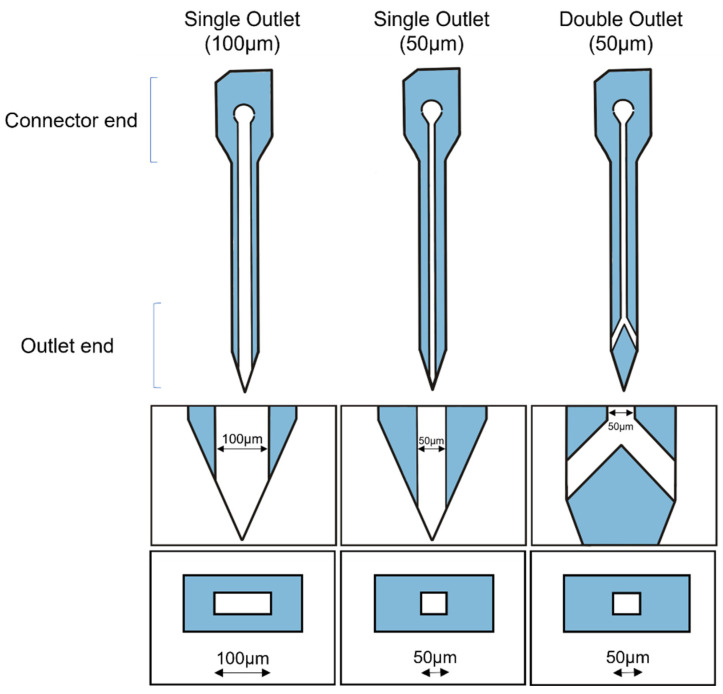
Single and branched probe designs with single and double outlets. The top row shows the overall design of a probe with 100 µm and 50 µm wide channels in either a single-outlet design or a double-outlet branched design. The middle row shows a close-up of the tips of the probes and their channels. The bottom row shows a cross-section of the probes and the microfluidic channels running through the shanks.

Three NC microfluidic neural probe designs were evaluated in vivo (Figure 3). Each microfluidic neural probe has a region for interfacing to off-probe drug sources (“connector end”) and a shank approximately 3 mm in length that terminates in a sharp tip. Fluid enters the microfluidic channel integrated within the NC probe through an inlet at the connector end and exits at one or two discrete outlets at or near the tip, respectively. Microfluidic channel widths of 50 µm and 100 µm were evaluated for the single-outlet devices, while only 50 µm channels were evaluated for the 2-outlet design. In addition, because the polymer used to create the NC absorbs some water, dissolved resveratrol molecules are able to diffuse through the NC structure, i.e., through the microfluidic walls along the microfluidic channel. Single outlet probes with 100 µm and 50 µm wide channels were named S100 and S50, respectively. Double outlet probes with 50 µm channels were named D50. The film fabrication methods were denoted with a hyphenated “-me” for mold-embossed, and “-m” for mold-only.

#### 2.3.3. Packaging

Custom 3D-printed microfluidic connectors facilitated interfacing the inlets of individual probes to PE-50 tubing (Braintree Scientific, Braintree, MA, USA), and Alzet osmotic pumps (model 2006; Alzet, Cupertino, CA, USA) to make a completed device [52]. The 3D-printed connectors were made using ABS-based plastic (VisiJet M3-X, 3D Systems, Rock Hill, SC, USA) with a wax base, as detailed in previous publications [52]. The probes were aligned to the connectors and then bonded by heating at 60 °C for 1 min and 30 s. Two-part epoxy was used to seal the joint between the PE-50 tubing and the 3D 3D-printed microfluidic connector. Packaged devices were sterilized using cold ethylene oxide gas, to be connected to the Alzet osmotic pumps in a sterile environment for in vivo implantation.

### 2.4. Benchtop Characterization

#### Flow Testing for Functionality Validation

**Figure 4:**
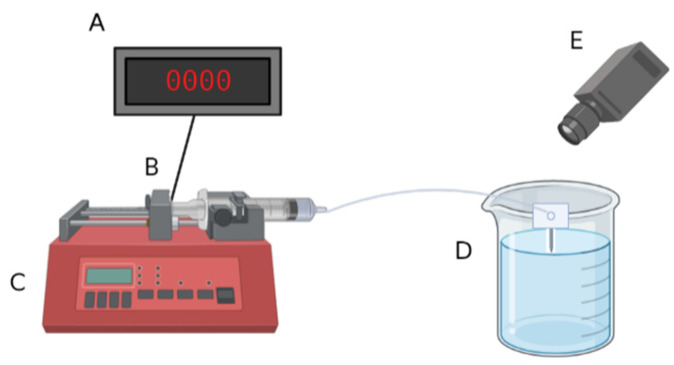
Schematic of setup for functionality validation. (**A**) A load cell readout is connected to a computer and records the force. (**B**) A load cell attached to the syringe pump detects force at the syringe plunger. (**C**) A syringe pump with a Luer-lok syringe filled with air. (**D**) A completed device connected to the syringe pump and submerged in a beaker of DI water. (**E**) A microscope camera to record process and detect bubbles at the probe tip.

The ability of packaged probes to transport fluids through the microfluidic channel was assessed by pushing air through the probe with a syringe controlled by a syringe pump, measuring the force on the syringe, and observing the appearance and location of air bubbles in the probes (Figure 4). Packaged probes were connected to a 3 mL Luer-Lok syringe filled with air by fitting the PE-50 tubing over 27-gauge blunt needles. The syringes were placed on a syringe pump, and the microfluidic devices were submerged in DI water. The syringe pump pushed air through the syringe, tubing, microfluidic connector, and the probe at 100 µL/h, which is within the range of common microdialysis flow rates (18–120 µL/h) [53]. The high flow rate during validation compared to the 0.15 µL/h flow rate in vivo (based on the rating of the osmotic pumps) was used to accelerate potential failure modes prior to implantation to ensure proper function in vivo. Devices were visually observed using a microscope camera, and the force on the syringe plunger was monitored using a load cell (SLB-25, Transducer Techniques, Temecula, CA, USA). Only probes that demonstrated fluid flow without leaks at the connector or along the probe shank were considered to have passed the flow test and were fit to advance to in vivo tests. Fluid path leaks, as indicated by air bubbles at the connector or along the probe shank, or clogs, as indicated by an applied syringe force > 3000 g, indicated that the devices were unsuitable for in vivo studies. Examples of properly functioning probes versus probes that failed because of breakage at the connector-probe interface are shown in Supplementary Materials Figure S1.

### 2.5. In Vivo Testing

Single-outlet probes with 100 µm-wide channels were connected and implanted into Sprague–Dawley rats for up to 4 weeks (N = 5), or with single-outlet (N = 3) or double-outlet (N = 4) 50 µm-wide channels for 6 weeks. Animals were implanted with packaged probes made using the mold-emboss method and connected to osmotic pumps with the flow of 30 µM solution of resveratrol in saline with a rate of 0.15 µL/h. The designs were compared to a control: a NC microfluidic probe cut to the same dimensions of the experimental probes (S50-me), but not connected to an osmotic pump (N = 4). The osmotic pump with the 0.15 µL/h flow rate was chosen by taking into account the therapeutic dose necessary of 30 µM, and the interstitial fluid (ISF) clearance rate [54,55,56,57,58]. The purpose of this study was to aid in determining the **best-performing** design to implement in future studies. The evaluation of “best-performing” will consider both the robustness of the device design and the in vivo histological response.

#### 2.5.1. Probe Priming

Packaged probes (Figure 7D) were sterilized using cold ethylene oxide gas. In a sterile environment, Alzet osmotic pump reservoirs (model 2006; Alzet, Cupertino, CA, USA) were weighed before and after filling with nominally 200 µL of a sterile solution of 30 µM trans-resveratrol (Mega Resveratrol, Danbury, CT, USA) in saline, to determine the initial volume of the solution. The pumps were then connected to the tubing of the packaged probes and placed in a 50 mL conical tube filled partly with sterile saline solution such that only the pump was submerged in the fluid. The conical tube was sealed and allowed to continue priming in an incubator at 37 °C for 60 h to ensure the solution was within the microfluidic channel prior to implantation.

#### 2.5.2. Animal Implantation

Surgical implantation in Sprague–Dawley rats followed our established protocols [13,36,59,60,61,62]. Briefly, rats were anaesthetized using 2.5% isoflurane until there was no response to a toe pinch. The area of surgery was shaved, and eye lubricant was applied to the rat. Meloxicam (1 mg/kg) and cefazolin (16 mg/kg) were administered subcutaneously to relieve pain and prevent infection, respectively. Marcaine (0.25%) was administered subcutaneously at the site of surgery for local anesthesia. Once the rat was prepared, it was mounted on a stereotaxic frame with flowing 2.0% isoflurane and 1.5 L/min oxygen, and the surgical site was sterilized with alternating scrubs of betadine and isopropyl alcohol. Vitals were monitored using a MouseSTAT Pulse Oximeter and Heart Rate Monitor (Kent Scientific Corp., Torrington, CT, USA).

The anesthetic plane was tested once again before the initial midline incision on the scalp, and the periosteum was cleaned off using cotton-tipped applicators to expose the skull. Hydrogen peroxide was applied to dehydrate the skull, and Vetbond (3M, St. Paul, MN, USA) was applied to prime the skull for the craniotomy to ensure smooth drilling. Using a 1.45 mm drill bit on a Kopf dental drill (David Kopf Instruments, Tujunga, CA, USA), craniotomies were made at 3 mm lateral and 3 mm anterior to the bregma. The drilling duration was pulsed to avoid the generation to thermal damage to the blood–brain barrier [63,64]. A subcutaneous pocket was made in the animal’s back about 7 cm posterior to account for the length of tubing and osmotic pump in preparation for implantation [65].

**Figure 5:**
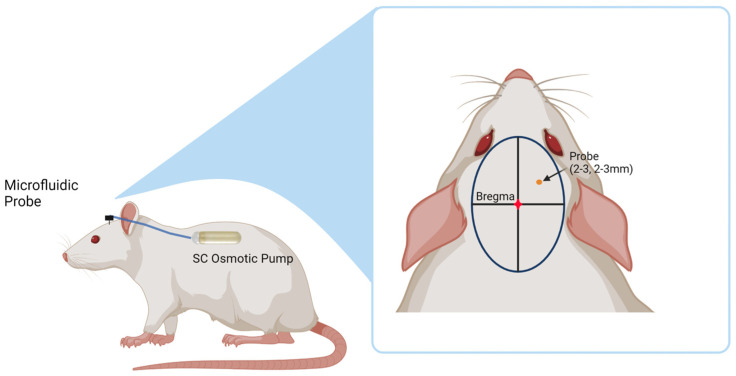
Diagram showing placement of the probe and osmotic pump in the rats. The probe is placed 2–3 mm anterior and lateral to the bregma. The osmotic pump is placed in a subcutaneous pocket in the mid-scapular region of the rat’s back. (Created with https://www.biorender.com/, accessed on 11 February 2023).

Primed microfluidic probes were secured using a custom holder attached to the stereotaxic frame. The probes were then lowered 3 mm into the motor cortex and the craniotomy was sealed with a layer of Kwik-Cast (World Precision Instruments, Sarasota, FL, USA) and Teets Cold Cure Dental Cement (A-M Systems, Sequim, WA, USA). The osmotic pump was placed into the subcutaneous pocket, taking care to lay the tubing flat. A headcap was made using dental cement to encompass the probe connector and part of the tubing to ensure secure attachment. The incision site was sutured closed, and the animals were monitored 5 days post-operatively to ensure proper healing. Figure 5 shows a diagram of the placement of both the probe and the osmotic pump in the animal. Animals were administered meloxicam (1 mg/kg) for pain once a day for two days, and cefazolin (16 mg/kg) as an antibiotic twice a day for one day post-implantation.

#### 2.5.3. Cardiac Perfusion

At the end of the study timepoint, the animals were sacrificed via transcardial perfusion. Intraperitoneal (IP) injections of ketamine (80 mg/kg) and xylazine (10 mg/kg) were administered to the animals and allowed to reach an aesthetic plane, which was confirmed with a toe pinch. A peristaltic pump (VWR International, Radnor, PA, USA) was used to pump 500–800 mL 1X PBS first, and then 300–500 mL 10% phosphate buffered formalin (Fisher Scientific, Hampton, NH, USA) at a rate of 8.3 mL/min. The brain was harvested, along with the skull attached to the headcap. Saline was used to gently wash explanted probes recovered post-mortem. The explanted osmotic pump reservoirs were also collected and stored.

#### 2.5.4. Imaging of Explanted Probes

Explanted probes were washed with 1X PBS + 0.1% triton briefly, and then incubated in DAPI to stain any cellular nuclei adhered to the probes. The probes were then dried and imaged using a confocal microscope (Nikon A1R HD Multiphoton System, Nikon Instruments Inc., Melville, NY, USA) to determine if there were any adhered cells on the surface of the probes or inside the channels. After fluorescent confocal microscopy, the probes were then placed under an optical microscope (Keyence, VHX-7000, Osaka, Japan) and imaged at high magnification.

#### 2.5.5. Explanted Osmotic Pump Analysis

Resveratrol solution remaining in the osmotic pumps at the given timepoint was removed from the explanted pump using 27 g needles and placed into 1 mL Eppendorf tubes, according to instructions from Alzet [65]. The initial drug volume in the osmotic pump was calculated using the weight difference before and after filling the pumps, and the density of the diluent solution (saline density = 1.0046 g/cm^3^) [65]. The tubes were spun in a centrifuge at 1000 rpm for 2 min to ensure all fluid was at the bottom of the tube. The fluid remaining was measured with a pipette (Finnipipette F2 2–20 µL and 20–200 µL). The remaining fluid was compared to the initial volume of fluid to determine cumulative delivery of resveratrol solution. To prevent evaporation of the fluid, all osmotic pumps were analyzed immediately after extraction. In all other steps, all containers were immediately capped to also prevent solution evaporation.

#### 2.5.6. Histology

Histology protocols for neuronal nuclei density (NeuN; mouse anti-neuronal nuclei, Millipore Sigma, Burlington, MA, USA), blood–brain barrier permeability (IgG; rabbit anti-immunoglobulin, Millipore Sigma, Burlington, MA, USA), immune cell activation (CD68; mouse anti-CD68, Millipore Sigma, Burlington, MA, USA), and glial cell activation (GFAP; rabbit anti-glial fibrillary acidic protein, Millipore Sigma, Burlington, MA, USA) were similar to those published in previous studies in our laboratory [13,36,66,67]. Briefly, slides were allowed to adjust to room temperature for 30 min, and OCT (Optimal Cutting Temperature) Compound Medium (Fisher Scientific, Hampton, NH, USA) was washed off with three washes of 1X PBS. Tissue was permeabilized with a 15 min incubation in 1X PBS + 0.1% triton solution for 15 min, and then blocked with a goat buffer solution for 1 h. After blocking, the tissue was incubated with primary antibodies overnight. The next day, primary antibodies were washed off with 1X PBS + 0.1% triton, and secondary antibodies were applied for 2 h. After incubation in secondary antibodies, the slides were washed three times in 1X PBS + 0.1% triton first, then three times with 1X PBS. A 0.5 mM copper sulfate solution was incubated on the slides for exactly ten minutes to decrease autofluorescence and background noise, and then washed off with three consecutive deionized (DI) water washes [68]. Slides were mounted with Fluoromount-G (Thermo Fisher Scientific, Waltham, MA, USA) and allowed to dry in a cool, dark area.

An automated immunohistochemistry stainer (Leica Bond Rx, Leica, Wetzlar, Germany) was used to stain for oxidized proteins (rabbit anti-Nitrotyrosine; Cayman Chemical, Ann Arbor, MI, USA). The slides were removed from −80 °C deep freezer and allowed to acclimate to room temperature in a humidity chamber for 30 min and washed with three consecutive 1X PBS washes prior to placing in the Bond Rx to remove OTC. The slides were then washed with the Bond Rx proprietary wash buffer and underwent epitope retrieval also using the company’s proprietary epitope retrieval solution. Peroxide buffer was applied and washed off, and primary antibodies were applied. After the primary were washed, the slides were taken out of the Bond Rx to be incubated in secondary antibody solution for two hours. Copper sulfate solution was applied for 10 min before washing with DI water and mounting with Fluoromount-G. The slides were dried in a cool, dark area before imaging.

Axioscan Z1 (Zeiss Inc., Oberkochen, Germany) with the 20X objective was used to fluorescently image the slides at the same exposure times for each fluorescent marker. The resulting images were subset to reduce file size and exported as a TIFF file for further analysis using a custom MATLAB algorithm called SECOND. In this software, the implant holes were marked and any artifacts were excluded before intensity analysis was conducted [69]. Another custom MATLAB algorithm called AfterNeuN was used to count neuronal nuclei density [67]. Both data in SECOND and AfterNeuN were normalized to background levels of intensity and density, respectively, in the 450–500 µm bin.

## 3. Results and Discussion

### 3.1. Stylus Profilometry and Optical Microscopy for Depth and Width

**Figure 6:**
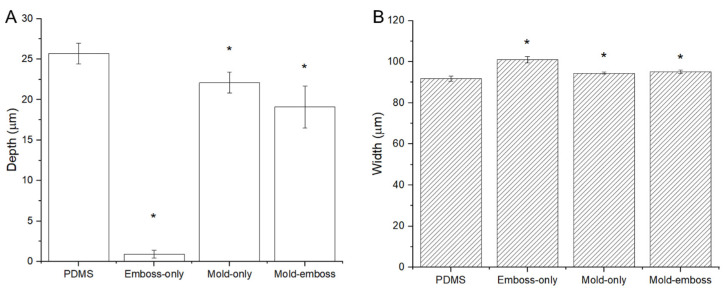
Stylus profilometry and optical imaging results for depth (**A**) and width (**B**) measurements of 100 µm-wide microfluidic channels in films fabricated using the mold-only method, embossed-only method, and mold-embossed method. The PDMS mold used was also measured using optical imaging. All methods were significantly different from both the PDMS mold and each other, distinguished with asterisks (*), in width and depth. *p* < 0.001.

Stylus profilometry measurements indicated that the depths of each NC channel layer fabrication method ranged from 3.5% to 86% of the height of the PDMS negative mold (Figure 6A). The embossed-only technique resulted in a very shallow channel imprint. Both the mold-only and mold-emboss films also had significantly different depths compared to the PDMS mold (*p* < 0.001) but were closer to the depth measurements of the mold. The mold-only films had the deepest channels after channel layer film fabrication. High-magnification optical microscopy imaging showed that the widths of the films resulting from each fabrication method ranged from 2.8% to 10% larger than the PDMS mold (Figure 6B). Deformation caused by the compression applied to the mold-emboss method is likely the cause of the reduction in channel depth compared to the channels from the mold-only method. The small width discrepancies resulted from differences in thermal expansion between PDMS and NC, as well as the deformation of the PDMS relief structure during compression. The emboss-only method was unable to produce channels of sufficient depth and accuracy. Though the results may be improved with increasing the temperature, pressure, or emboss time, these conditions may also cause degradation of the material. Therefore, the emboss-only method was not considered in further characterization experiments.

### 3.2. Mechanical Properties of As-Cast and Pressed Materials

Tensile testing was used to characterize differences in mechanical properties that result from the embossing step. Mechanical analysis of planar regions from the as-cast and pressed films, corresponding to the mold-only and mold-embossed microfluidic channels respectively, indicated that pressing increased the Young’s modulus significantly (Table 1). The pressed films also have a significantly higher stress at maximum load than mold-only films. A representative stress-strain plot of samples from as-cast and pressed films can be found in Figure S1. The higher Young’s modulus, strength, and strain-to-break of the pressed materials indicate that the mold-emboss fabrication methods allows for more robust implants that can be inserted into brain tissue with smaller cross-sectional dimensions than would be possible with the mold-only method. Pressing and embossing may have had the effects of aligning the cellulose nanocrystals and increasing the density, allowing for more efficient hydrogen bonding in the dry films, and increasing the film stiffness compared to the as-cast films [26,70].

### 3.3. Fabricated Devices

**Figure 7:**
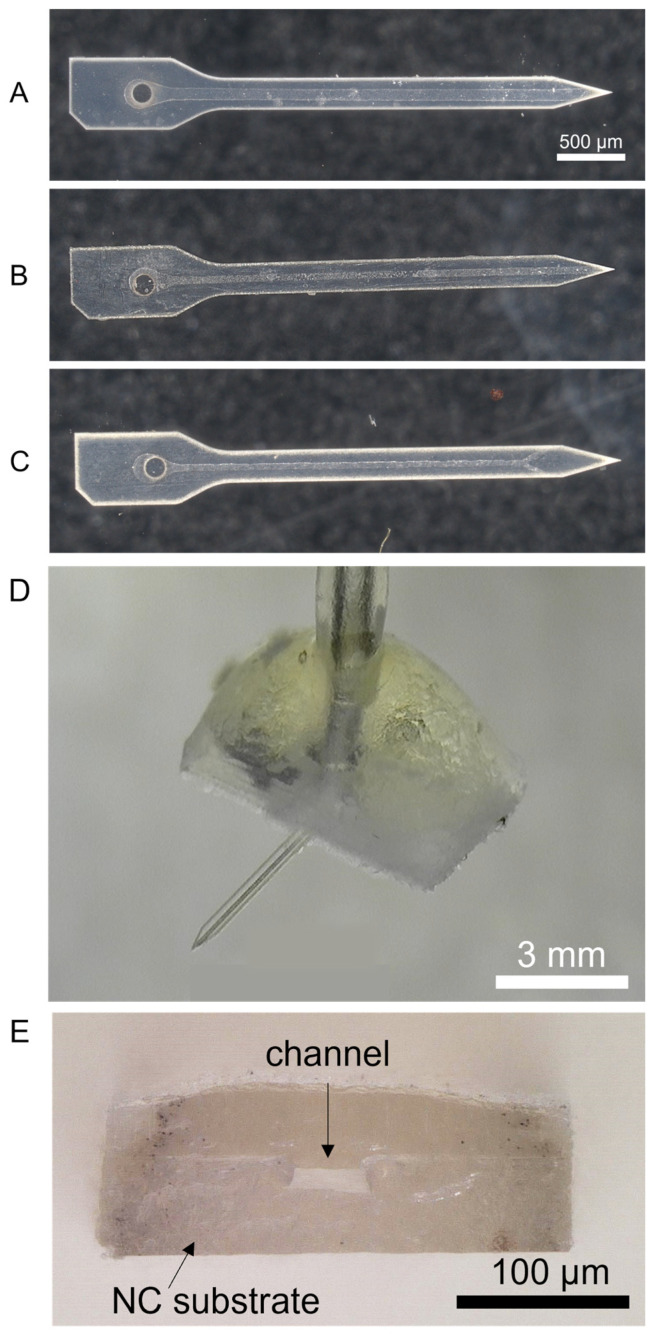
(**A**) Image of a mold-embossed probe with a 100 µm-wide (S100-me) good channel before packaging. (**B**) Image of a mold-embossed probe with a 50 µm-wide (S50-me) good channel pre-packaging. (**C**) Image of a mold-embossed probe with 50 µm-wide channels and two outlets (D50-me) with a good channel pre-packaging. Scale bar = 500 µm. (**D**) Image of a representative completed device with connector and tubing attached. Scale bar = 3 mm. (**E**) Cross-section image of a probe with a 50 µm-wide channel. Scale bar = 100 µm.

Individual probes were imaged to inspect and ensure distinct, continuous channels and proper alignment of the cover and channel layers before packaging with a connector and tubing. Figure 7A–C shows representative images of the three probe designs. The probes were then packaged with the connector and tubing, resulting in a completed device (Figure 7D) suitable for testing. A cross-section of one of the devices with a 50 µm-wide channel is shown in Figure 7E.

#### Cross-Sectional Analysis of Thermally Bonded Films

Cross-sections of individual microfluidic probes showed that after thermal bonding, the channels in the mold-only probes were deformed with a channel height of only ~25–50% of the channel depth prior to bonding (Figure S2A). Mold-embossed probes with 100 µm-wide (S100-me) and 50 µm-wide (S50-me) channels maintained the channel shape (Figure S2B and Figure 7E). Overall, the yield was the highest for the mold-emboss probes with 50 µm-wide channels. The microfluidic probes from the higher modulus mold-embossed films produced more structurally stable devices that were better able to maintain the microfluidic channel shape.

### 3.4. Flow Testing for Functionality

Functionality testing indicated that the mold-embossed microfluidic channel fabrication method yielded a high percentage of devices suitable for implantation. Only 24% of 21 mold-only probes successfully allowed for syringe-applied air-flow through the channel to the outlet, while 58% of 36 mold-embossed probes were successful. Successful fluid flow was defined as having air bubbles appear at the microfluidic channel outlet (Figure S3), and only at the microfluidic channel outlet, with less than 1000 g of force applied to the syringe. The average force required for mold-only probes to demonstrate successful fluid flow was 347 g of force, while the mold-embossed probes only required 136 g of force. If the force required was higher than 1000 g, the probe was assumed to have an occlusion and the data were excluded from the analysis.

Table 2 details the forces required to push air through the successful devices using the load cell-instrumented syringe pump during functionality tests. The mold-only group (S100-m) exhibited higher flow force and variability compared to the mold-embossed groups. Varying channel widths and designs did not have a significant difference compared to the material processing variable. However, it is worth noting that the probes with 50 µm-wide channels had a higher flow force than the probes with 100 µm-wide channels. Adding a second outlet decreased the flow force such that the mold-embossed probes with 50 µm-wide channels and two outlets (D50-me) had the lowest average flow force. Flow force analysis for all probes regardless of success or failure can be found in Table S1.

The flow force is related to the fluidic resistance of the channels and is expected to increase with a decrease in cross-sectional channel area. The higher force for the S100-m probes is consistent with the cross-sectional analyses (Figure S2) that indicated the channels deformed and partially collapsed during the bonding step. The higher force for the S50-me probes compared to the S100-me probes is expected based on the decrease in channel area. Increasing the number of outlets effectively increased the cross-sectional area of the microfluidic channel at the branch point, therefore reducing the force required to push air through the probe.

Based upon the results of the cross-sectional images and the functionality testing, it was determined that the mold-embossed method produced the highest yield and most consistent microfluidic probe devices. Therefore, in vivo studies were performed using only microfluidic probes made using the mold-embossed method.

### 3.5. In Vivo Fluid Flow Functionality

Pilot in vivo studies showed that S100-me microfluidic channels collapsed after implantation (Figure S4). Xue et al. explored similar collapse in polymer microfluidic channels and what aspects could be changed to mitigate this event [71]. Investigation showed that the normalized work of adhesion for one-sided collapse must be less than 36 to prevent channel collapse (${\bar{\gamma }}_{channel}<36)$ [71]. Based on the material properties of the NC material in a wet state (5 MPa), and PVAc work of adhesion ($\gamma_{PVAc}=37\;\mathrm{mJ}/m^{2}$), calculations were performed to determine the maximum channel width that would avoid channel collapse [71,72,73]. We found that at channel widths larger than 58 µm, the normalized work of adhesion increased above 36, indicating future designs must have channels smaller than 58 µm. As a result, new probe designs were created with 50 µm-wide channels (S50-me), and a branching design (D50-me) was introduced with outlet ports placed on the side of the shank.

After the 6-week implant duration for the S50-me and D50-me probes, the remaining resveratrol solution in the osmotic pump was measured and compared to the starting volume. The measured average starting volumes for the osmotic pumps implanted in 6-week implant duration animals were 173 ± 34 µL. After the course of the study, the measured average cumulative elution volume of the resveratrol solution was 155 ± 40 µL. The rate of fluid flow calculated over the total elution time duration of the osmotic pumps resulted in an average flow rate of 0.15 ± 0.04 µL/h that matched the manufacturer’s specification. Further investigation showed that S50-me probes had an average total elution volume of 132 ± 31 µL, and the D50-me probes had an average total elution volume of 186 ± 40 µL. With a *p* = 0.054, the differences are not statistically significant, but it is worth noting that the double outlet probes seemed to have higher elution volumes than single outlet probe designs. On ex vivo inspection for signs of occlusion, some debris were apparent in DAPI-stained S50-me probes, but no debris were seen for the double-outlet D50-me probes (Figures S4–S6). This was indicative that brain tissue going into the outlet during insertion is less when the outlet is placed on the sides and angled as in the D50 design, compared to a straight outlet at the tip in the S100 and S50 designs. The debris likely contributed to a slower average delivery rate. This study indicated that these microfluidic neural probes were able to continuously deliver resveratrol over a 6-week duration.

### 3.6. Histology from Animals Implanted with Modified Design Probes

**Figure 8:**
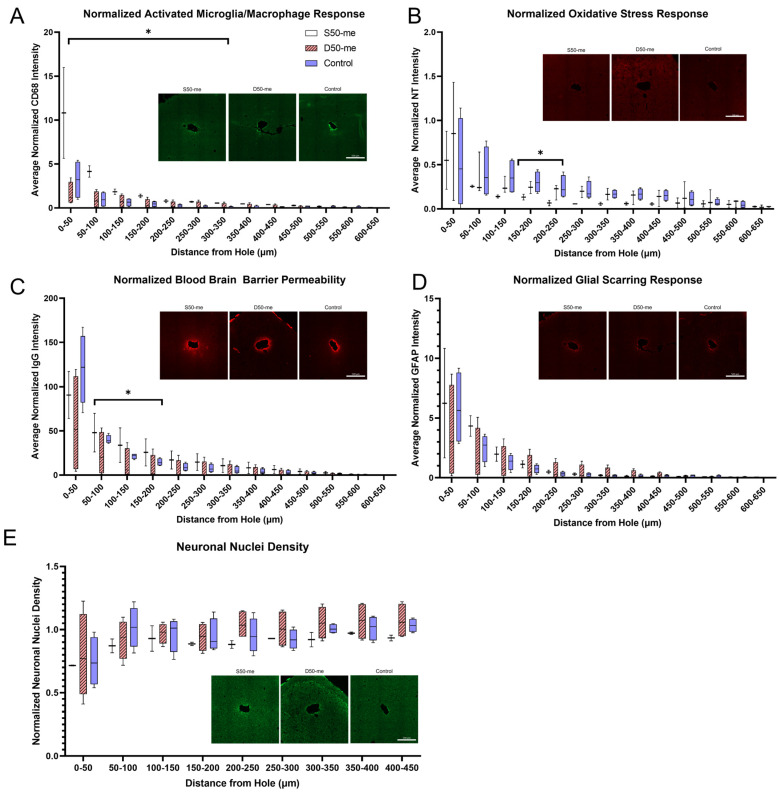
Histological response to implanted probes using 50 µm–wide channels with a single outlet (N = 3), double outlet (N = 4), and control with no resveratrol delivery (N = 4). (**A**) Macrophage/monocyte response, (**B**) blood–brain barrier permeability, (**C**) oxidative stress response, (**D**) glial scarring response, and (**E**) neuronal nuclei density were evaluated to determine which of the probe designs had the best tissue response. *p* < 0.05. Significance is denoted by the asterisk (*) in the plots.

Histological analysis (Figure 8) showed that the single outlet design (S50-me) had increased macrophage/monocyte response from 0–300 µm distances from the hole compared to controls, which had no drug or diluent delivered. In comparison, the double outlet design D50-me did not have a significant difference in macrophage/monocyte activation compared to controls. The higher macrophage/monocyte activation for S50-me compared to controls indicated that the design of the probe contributed to higher immune cell reactivity while also delivering an antioxidant, compared to the control that was not delivering any drug. This behavior is not beneficial for tissue health. It is possible that the fluid flowing from the microfluidic devices slightly increased the inflammatory response to the implant compared to the control devices. Additionally, the 40% increased dose of resveratrol associated with the D50-me compared to the S50-me could have been enough to compensate for the agitation associated with fluid release into the tissue space.

Meanwhile, the single-outlet design S50-me had decreased oxidative stress response compared to controls at 150–250 µm distances, as you would anticipate with increased efficiency of the delivery of antioxidants to the device-tissue interface [28,36]. Although it seemed as if the D50-me design also may have had a decreased oxidative stress response, the difference was not statistically significant—which may be a result of the low animal numbers. The histological responses are interesting, as although the S50-me probes had higher levels of activated immune cells and BBB permeability, it also had the lowest oxidative stress levels. Again, these confounding results could have been due to the decreased overall release of resveratrol in the S50-me compared to the D50-me, as well as a differential displacement of the resveratrol associated with the various designs.

There was no significant difference between the two design responses compared to the control for glial scarring, blood–brain barrier (BBB) permeability, and neuronal nuclei density. Other resveratrol delivery studies performed in the laboratory have shown improvements in neuronal density, but not for glial scarring [13,16,29]. Antioxidant delivery was expected to result in a decrease of these inflammation markers [74]. However, there have been studies that also show antioxidant delivery did not significantly change the neuroinflammatory tissue response [59]. This study also looked at oxidative stress, which is important in cellular health. The delivery of resveratrol resulted in decreased oxidative stress, consistent with previous studies in the laboratory [36]. Resveratrol’s mechanism of action is twofold: quenching existing reactive oxygen species (ROS) and upregulating endogenous enzymes to prevent future production of ROS [16,75,76]. Therefore, even if there are activated immune cells, oxidative stress may be mitigated through endogenous enzymes. However, the physical presence of the probe in situ still results in the activation of glial and immune cells.

Explanted probes that were stained with DAPI observed that the side placement of the outlets decreased cellular debris inside of the channels (Figure S5), whereas the single outlet design had cellular debris regardless of channel size. While the histology results are based on a relatively few number of implants, the general trend of lower inflammatory markers in D50-me rather than S50-me compared to the controls supports our decision of moving forward with this design in future studies. The combined results of histology, explanted pump analysis, and design considerations indicate that not only are the microfluidic channel integrated probes viable for chronic implantation and continuous drug delivery, but the D50-me design is also the best suited of the designs tested here for future applications.

The primary objective of this work was to optimize fabrication methods and design for microfluidic channels integrated into a mechanically adaptive polymer nanocomposite. The optimized fabrication method combined micromolding and embossing to form the channel layer, which was then bonded to a cover layer. Though the mold-only process initially produced channels that better matched the inverse of the PDMS mold, the process of soaking and thermally bonding to the cover layers resulted in considerable deformation and collapsing of the channels. Additionally, mold-only devices required more than twice the force to push air through the channel and had a lower “success” yield compared to the mold-emboss process. Material testing confirmed that the process of embossing yielded a higher modulus compared to mold-only films, which contributed to the improved stability of the mold-embossed devices in terms of the ability to maintain the channel geometry during the thermal bonding step. The results of the characterization determined that the best method for fabricating the probes was the mold-embossed method.

The in vivo study showed that these probes, when connected to osmotic pumps, were able to deliver resveratrol solution continuously and consistently to the site of implantation over the course of 6 weeks. Additionally, the double-outlet branching design showed the best performance in histology neuroinflammation metrics compared to the single outlet design, supporting such designs for future full studies. Our design allows multiple avenues of drug delivery to the site of implantation by having multiple outlets and a permeable mechanically adaptive material that can diffuse drug through the substrate. Other designs in the field have either used stiff substrates with open ports on the tip, or flexible substrates with open ports [45,47]. Using stiff substrates may exacerbate the inflammatory response, while relying on a fixed number of ports to deliver therapeutics may prove ineffective if there is occlusion of the ports due to cellular debris during insertion. Our design mitigates such concerns since the material is mechanically adaptive and permeable to therapeutic drugs.

## 4. Conclusions

The mechanically adaptive microfluidic neural probe optimized and characterized in this paper establishes a platform wherein both the drug delivered and the time course of delivery can be altered to fit a patient’s needs. The mechanically softening aspect of the probe substrate aims to reduce neural inflammation, with the goal of producing IMEs with improved performance at chronic time points. The additional delivery of a drug such as an antioxidant may further improve IME performance and longevity. However, the drug may also be swapped out for any therapeutic designed to improve brain tissue health and function. The flexibility of our design lends itself ‘flexible’ to the discovery of additional targets with the recent use of more sophisticated analysis methods of the neuroinflammatory response to IMEs [13,77,78,79,80,81,82]. The ability for this probe to be a platform device makes it a powerful tool that may help improve patient quality of life. Future studies will aim to first validate chronic efficacy in a long-term study of the effect of utilizing both soft substrates and antioxidants on neural inflammation. The next step would be to integrate recording electrodes and miniaturize the probes to determine the extent of IME. 

Performance enhancement given these conditions, in comparison to the industry standard of rigid silicon implants. Previous studies have demonstrated the ability to integrate recording electrodes that can record up to 16 weeks, with dimensions better matching rigid silicon NeuroNexus probes [32]. Therefore, future studies will be able to utilize the background completed for both microfluidic and recording capabilities to develop and determine performance abilities compared to the industry standard.

## Figures and Tables

**Table 1:** Material characteristics of as-cast planar films compared to pressed planar films, corresponding to the conditions used to produce the mold-only and mold-embossed microfluidic channels, respectively. The pressed films have a significantly higher modulus than the as-cast films, *p* = 0.01. Significance is marked by the asterisk (*). The stress at the maximum load was significantly higher in pressed films compared to as-cast films, *p* = 0.01. The strain at the maximum load was not found to be statistically different.

Method	Young’s Modulus (MPa)	Stress at Max Load (MPa)	Strain at Max Load (%)
As-cast (mold-only)	2428 ± 151 *	23 ± 7 *	0.21 ± 0.4
Pressed (mold-emboss)	3331 ± 438 *	41 ± 5 *	0.49 ± 0.1

**Table 2:** Average ± standard deviation, maximum, and minimum observed fluid force (reported in grams, g) exerted to transport fluid through microfluidic channels in probes made using mold-only and mold-embossed methods with either single-outlet or double-outlet. Mold-only 100 µm channel single outlet probes (S100-m) had significantly higher forces than the mold-embossed 100 µm single outlet (S100-me), mold-embossed 50 µm channel single outlet (S50-me), and mold-embossed 50 µm channel double outlet (D50-me). The asterisk on the S100-m indicates significance with *p* < 0.05. The table details fluidic forces recorded for only successful probes.

	S100-m * (g)	S100-me (g)	S50-me (g)	D50-me (g)
Average	347 ± 114	136 ± 60	180 ± 70	128 ± 41
Maximum	486	356	336	174
Minimum	207	78	89	81

## Data Availability

Data available upon request due to ethical restrictions.

## Supplementary Materials

The following supporting information can be downloaded at: https://www.mdpi.com/article/10.3390/mi14051015/s1, Figure S1: Representative mechanical properties of as-cast and pressed materials; Figure S2: Cross-sectional analysis of thermally bonded films; Figure S3: Images of probe functionality testing; Table S1: Functionality testing pressure analysis; Figure S4: Explanted S100-me probe showing a collapsed channel; Figure S5: Confocal 3D DAPI images of explanted S100-me probes; Figure S6: Images of explanted D50-me probes.

## References

[1] Shih J.J., Krusienski D.J., Wolpaw J.R. (2012). Brain-Computer Interfaces in Medicine. Mayo Clin. Proc..

[2] Association for Computing Machinery (2007). Special Interest Group on Accessible Computing. Proceedings of the ASSETS ’07 Ninth International ACM SIGACCESS Conference on Computers and Accessibility.

[3] Fetz E., Finocchio D. (1975). Correlations between activity of motor cortex cells and arm muscles during operantly conditioned response patterns. Exp. Brain Res..

[4] Schmidt E.M. (1980). Single neuron recording from motor cortex as a possible source of signals for control of external devices. Ann. Biomed. Eng..

[5] Serruya M.D., Hatsopoulos N.G., Paninski L., Fellows M.R., Donoghue J.P. (2002). Instant neural control of a movement signal. Nature.

[6] Ajiboye A.B., Willett F.R., Young D.R., Memberg W.D., Murphy A.B.A., Miller J.P., Walter B.L., Sweet A.J., Hoyen H.A., Keith M.W. (2017). Restoration of reaching and grasping movements through brain-controlled muscle stimulation in a person with tetraplegia: A proof-of-concept demonstration. Lancet.

[7] Hochberg L.R., Serruya M.D., Friehs G.M., Mukand J.A., Saleh M., Caplan A.H., Branner A., Chen D., Penn R.D., Donoghue J.P. (2006). Neuronal ensemble control of prosthetic devices by a human with tetraplegia. Nature.

[8] Buzsáki G. (2004). Large-scale recording of neuronal ensembles. Nat. Neurosci..

[9] Ortiz-Rosario A., Adeli H. (2013). Brain-computer interface technologies: From signal to action. Rev. Neurosci..

[10] Barrese J.C., Rao N., Paroo K., Triebwasser C., Vargas-Irwin C., Franquemont L., Donoghue J.P. (2013). Failure mode analysis of silicon-based intracortical microelectrode arrays in non-human primates. J. Neural Eng..

[11] Salatino J.W., Ludwig K.A., Kozai T.D.Y., Purcell E.K. (2017). Glial responses to implanted electrodes in the brain. Nat. Biomed. Eng..

[12] Takmakov P., Ruda K., Phillips K.S., Isayeva I.S., Krauthamer V., Welle C.G. (2015). Rapid evaluation of the durability of cortical neural implants using accelerated aging with reactive oxygen species. J. Neural Eng..

[13] Ereifej E.S., Rial G.M., Hermann J.K., Smith C.S., Meade S.M., Rayyan J.M., Chen K., Feng H., Capadona J.R. (2018). Implantation of Neural Probes in the Brain Elicits Oxidative Stress. Front. Bioeng. Biotechnol..

[14] Jorfi M., Skousen J.L., Weder C., Capadona J.R. (2015). Progress towards biocompatible intracortical microelectrodes for neural interfacing applications. J. Neural Eng..

[15] Ludwig K.A., Miriani R.M., Langhals N.B., Joseph M.D., Anderson D.J., Kipke D.R., Allen B.D., Moore-Kochlacs C., Bernstein J.G., Kinney J.P. (2009). Using a Common Average Reference to Improve Cortical Neuron Recordings From Microelectrode Arrays. J. Neurophysiol..

[16] Potter K.A., Buck A.C., Self W.K., Callanan M.E., Sunil S., Capadona J.R. (2013). The effect of resveratrol on neurodegeneration and blood brain barrier stability surrounding intracortical microelectrodes. Biomaterials.

[17] Golabchi A., Wu B., Li X., Carlisle D.L., Kozai T.D., Friedlander R.M., Cui X.T. (2018). Melatonin improves quality and longevity of chronic neural recording. Biomaterials.

[18] Bickford P.C., Gould T., Briederick L., Chadman K., Pollock A., Young D., Shukitt-Hale B., Joseph J. (2000). Antioxidant-rich diets improve cerebellar physiology and motor learning in aged rats. Brain Res..

[19] Zhang H., Schools G.P., Lei T., Wang W., Kimelberg H.K., Zhou M. (2008). Resveratrol attenuates early pyramidal neuron excitability impairment and death in acute rat hippocampal slices caused by oxygen-glucose deprivation. Exp. Neurol..

[20] Wu C., Gopal K.V., Moore E.J., Gross G.W. (2014). Antioxidants l-carnitine and d-methionine modulate neuronal activity through GABAergic inhibition. J. Neural Transm..

[21] Singh M., Arseneault M., Sanderson T., Murthy V., Ramassamy C. (2008). Challenges for Research on Polyphenols from Foods in Alzheimer’s Disease: Bioavailability, Metabolism, and Cellular and Molecular Mechanisms. J. Agric. Food Chem..

[22] Scalbert A., Williamson G. (2000). Dietary Intake and Bioavailability of Polyphenols. J. Nutr..

[23] Walle T., Hsieh F., DeLegge M.H., Oatis J.E., Walle U.K. (2004). High absorption but very low bioavailability of oral resveratrol in humans. Drug Metab. Dispos..

[24] Hewlings S.J., Kalman D.S. (2017). Curcumin: A Review of Its Effects on Human Health. Foods.

[25] Walle T. (2011). Bioavailability of resveratrol. Ann. N. Y. Acad. Sci..

[26] Capadona J.R., Shanmuganathan K., Tyler D.J., Rowan S.J., Weder C. (2008). Stimuli-Responsive Polymer Nanocomposites Inspired by the Sea Cucumber Dermis. Science.

[27] Harris J.P., E Hess A., Rowan S.J., Weder C., Zorman A.C., Tyler D.J., Capadona J.R. (2011). In vivo deployment of mechanically adaptive nanocomposites for intracortical microelectrodes. J. Neural Eng..

[28] Nguyen J.K., Park D.J., Skousen J.L., E Hess-Dunning A., Tyler D.J., Rowan S.J., Weder C., Capadona J.R. (2014). Mechanically-compliant intracortical implants reduce the neuroinflammatory response. J. Neural Eng..

[29] Nguyen J.K., Jorfi M., Buchanan K.L., Park D.J., Foster J., Tyler D., Rowan S., Weder C., Capadona J.R. (2016). Influence of resveratrol release on the tissue response to mechanically adaptive cortical implants. Acta Biomater..

[30] Hess E.A., Capadona J.R., Shanmuganathan K., Hsu L., Rowan S.J., Weder C., Tyler D.J., Zorman A.C. (2011). Development of a stimuli-responsive polymer nanocomposite toward biologically optimized, MEMS-based neural probes. J. Micromechanics Microengineering.

[31] Harris J.P., Capadona J.R., Miller R.H., Healy B.C., Shanmuganathan K., Rowan S., Weder C., Tyler D.J. (2011). Mechanically adaptive intracortical implants improve the proximity of neuronal cell bodies. J. Neural Eng..

[32] Hess-Dunning A., Tyler D.J. (2018). A Mechanically-Adaptive Polymer Nanocomposite-Based Intracortical Probe and Package for Chronic Neural Recording. Micromachines.

[33] Sridharan A., Nguyen J.K., Capadona J.R., Muthuswamy J. (2015). Compliant intracortical implants reduce strains and strain rates in brain tissue in vivo. J. Neural Eng..

[34] Gilletti A., Muthuswamy J. (2006). Brain micromotion around implants in the rodent somatosensory cortex. J. Neural Eng..

[35] Jorfi M., Potter K.A., Nguyen J.K., Hess-Dunning A.E., Foster E.J., Capadona J.R., Weder C. Mechanically adaptive materials for intracortical implants. Proceedings of the International IEEE/EMBS Conference on Neural Engineering, NER, IEEE Computer Society.

[36] Kim Y., Ereifej E.S., Schwartzman W.E., Meade S.M., Chen K., Rayyan J., Feng H., Aluri V., Mueller N.N., Bhambra R. (2021). Investigation of the Feasibility of Ventricular Delivery of Resveratrol to the Microelectrode Tissue Interface. Micromachines.

[37] Shin H., Lee H.J., Chae U., Kim H., Kim J., Choi N., Woo J., Cho Y., Lee C.J., Yoon E.-S. (2015). Neural probes with multi-drug delivery capability. Lab A Chip.

[38] Pongrácz A., Fekete Z., Márton G., Bérces Z., Ulbert I., Fürjes P. (2013). Deep-brain silicon multielectrodes for simultaneous in vivo neural recording and drug delivery. Sens. Actuators B Chem..

[39] Rohatgi P., Langhals N.B., Kipke D.R., Patil P.G. (2009). In vivo performance of a microelectrode neural probe with integrated drug delivery. Neurosurg. Focus.

[40] Lee H.J., Son Y., Kim J., Lee C.J., Yoon E.-S., Cho I.-J. (2015). A multichannel neural probe with embedded microfluidic channels for simultaneous in vivo neural recording and drug delivery. Lab A Chip.

[41] Takeuchi S., Ziegler D., Yoshida Y., Mabuchi K., Suzuki T. (2005). Parylene flexible neural probes integrated with microfluidic channels. Lab A Chip.

[42] Wen X., Wang B., Huang S., Liu T., Lee M.-S., Chung P.-S., Chow Y.T., Huang I.-W., Monbouquette H.G., Maidment N.T. (2019). Flexible, multifunctional neural probe with liquid metal enabled, ultra-large tunable stiffness for deep-brain chemical sensing and agent delivery. Biosens. Bioelectron..

[43] Gao K., Li G., Liao L., Cheng J., Zhao J., Xu Y. (2013). Fabrication of flexible microelectrode arrays integrated with microfluidic channels for stable neural interfaces. Sens. Actuators A Phys..

[44] Kim A.A., Kustanovich K., Baratian D., Ainla A., Shaali M., Jeffries G.D.M., Jesorka A. (2017). SU-8 free-standing microfluidic probes. Biomicrofluidics.

[45] Altuna A., Bellistri E., Cid E., Aivar P., Gal B., Berganzo J., Gabriel G., Guimerà A., Villa R., Fernández L.J. (2013). SU-8 based microprobes for simultaneous neural depth recording and drug delivery in the brain. Lab A Chip.

[46] Jeong J.-W., McCall J.G., Shin G., Zhang Y., Al-Hasani R., Kim M., Li S., Sim J.Y., Jang K.-I., Shi Y. (2015). Wireless Optofluidic Systems for Programmable In Vivo Pharmacology and Optogenetics. Cell.

[47] Liu B., Kim E., Meggo A., Gandhi S., Luo H., Kallakuri S., Xu Y., Zhang J. (2017). Enhanced biocompatibility of neural probes by integrating microstructures and delivering anti-inflammatory agents via microfluidic channels. J. Neural Eng..

[48] Kim Y., Mueller N., Schwartzman W., Aluri V., Herried A., Capadona J.R., Hess-Dunning A. Hybrid Fabrication Method for Microfluidic Channels Within a Polymer Nanocomposite for Neural Interfacing Applications. Proceedings of the 21st International Conference on Solid-State Sensors, Actuators and Microsystems, TRANSDUCERS 2021.

[49] Rodrigues R.O., Lima R., Gomes H.T., Silva A.M.T. (2015). Polymer microfluidic devices: An overview of fabrication methods. U. Porto J. Eng..

[50] Capadona J., Shanmuganathan K., Trittschuh S., Seidel S., Rowan S., Weder C. (2009). Polymer Nanocomposites with Nanowhiskers Isolated from Microcrystalline Cellulose. Biomacromolecules.

[51] Berg O.V.D., Capadona J.R., Weder C. (2007). Preparation of Homogeneous Dispersions of Tunicate Cellulose Whiskers in Organic Solvents. Biomacromolecules.

[52] Szabo E., Hess-Dunning A. (2021). Irreversible, self-aligned microfluidic packaging for chronic implant applications. J. Micromechanics Microengineering.

[53] Hammarlund-Udenaes M. (2017). Microdialysis as an Important Technique in Systems Pharmacology—A Historical and Methodological Review. AAPS J..

[54] Mink J.W., Blumenschine R.J., Adams D.B. (1981). Ratio of central nervous system to body metabolism in vertebrates: Its constancy and functional basis. Am. J. Physiol-Regul. Integr. Comp. Physiol..

[55] Nieuwenhuys R., Donkelaar H.J.T., Nicholson C. (1998). The Central Nervous System of Vertebrates.

[56] Hammarlund-Udenaes M., Fridén M., Syvänen S., Gupta A. (2007). On The Rate and Extent of Drug Delivery to the Brain. Pharm. Res..

[57] Cserr H.F., Cooper D.N., Suri P.K., Patlak C.S. (1981). Efflux of radiolabeled polyethylene glycols and albumin from rat brain. Am. J. Physiol. Physiol..

[58] Szentistvanyi I., Patlak C.S., Ellis R.A., Cserr H.F. (1984). Drainage of interstitial fluid from different regions of rat brain. Am. J. Physiol. Physiol..

[59] Ziemba A.M., Woodson M.C.C., Funnell J.L., Wich D., Balouch B., Rende D., Amato D.N., Bao J., Oprea I., Cao D. (2022). Development of a Slow-Degrading Polymerized Curcumin Coating for Intracortical Microelectrodes. ACS Appl. Bio Mater..

[60] Haley R.M., Zuckerman S.T., Dakhlallah H., Capadona J.R., von Recum H.A., Ereifej E.S. (2020). Resveratrol Delivery from Implanted Cyclodextrin Polymers Provides Sustained Antioxidant Effect on Implanted Neural Probes. Int. J. Mol. Sci..

[61] Shoffstall A.J., Ecker M., Danda V., Joshi-Imre A., Stiller A., Yu M., Paiz J.E., Mancuso E., Bedell H.W., Voit W.E. (2018). Characterization of the Neuroinflammatory Response to Thiol-ene Shape Memory Polymer Coated Intracortical Microelectrodes. Micromachines.

[62] Ereifej E.S., Smith C.S., Meade S.M., Chen K., Feng H., Capadona J.R. (2018). The Neuroinflammatory Response to Nanopatterning Parallel Grooves into the Surface Structure of Intracortical Microelectrodes. Adv. Funct. Mater..

[63] Shoffstall A.J., Paiz J., Miller D.M., Rial G.M., Willis M.T., Menendez D.M., Hostler S.R., Capadona J.R. (2018). Potential for thermal damage to the blood–brain barrier during craniotomy: Implications for intracortical recording microelectrodes. J. Neural Eng..

[64] Hoeferlin G.F., Menendez D.M., Krebs O.K., Capadona J.R., Shoffstall A.J. (2022). Assessment of Thermal Damage from Robot-Drilled Craniotomy for Cranial Window Surgery in Mice. J. Vis. Exp..

[65] Durect Corporation ALZET Links. https://www.alzet.com/resources/links/.

[66] Potter K.A., Buck A.C., Self W.K., Capadona J.R. (2012). Stab injury and device implantation within the brain results in inversely multiphasic neuroinflammatory and neurodegenerative responses. J. Neural Eng..

[67] Potter-Baker K.A., Ravikumar M., Burke A.A., Meador W.D., Householder K.T., Buck A.C., Sunil S., Stewart W.G., Anna J.P., Tomaszewski W.H. (2014). A comparison of neuroinflammation to implanted microelectrodes in rat and mouse models. Biomaterials.

[68] Potter K.A., Simon J.S., Velagapudi B., Capadona J.R. (2012). Reduction of autofluorescence at the microelectrode–cortical tissue interface improves antibody detection. J. Neurosci. Methods.

[69] Goss-Varley M., Dona K.R., McMahon J.A., Shoffstall A.J., Ereifej E.S., Lindner S.C., Capadona J.R. (2017). Microelectrode implantation in motor cortex causes fine motor deficit: Implications on potential considerations to Brain Computer Interfacing and Human Augmentation. Sci. Rep..

[70] Rusli R., Shanmuganathan K., Rowan S.J., Weder C., Eichhorn S.J. (2010). Stress-Transfer in Anisotropic and Environmentally Adaptive Cellulose Whisker Nanocomposites. Biomacromolecules.

[71] Xue Y., Kang D., Ma Y., Feng X., Rogers J.A., Huang Y. (2017). Collapse of microfluidic channels/reservoirs in thin, soft epidermal devices. Extreme Mech. Lett..

[72] Kovačević V., Leskovac M., Blagojević S.L., Vrsaljko D. (2005). Complex Adhesion Effects of Inorganic Nanofillersvs Microfillers in Polymer Composites. Macromol. Symp..

[73] Shanmuganathan K., Capadona J.R., Rowan S.J., Weder C. (2010). Biomimetic mechanically adaptive nanocomposites. Prog. Polym. Sci..

[74] Potter K.A., Jorfi M., Householder K.T., Foster E.J., Weder C., Capadona J.R. (2014). Curcumin-releasing mechanically adaptive intracortical implants improve the proximal neuronal density and blood&ndash;brain barrier stability. Acta Biomater..

[75] Caillaud M., Chantemargue B., Richard L., Vignaud L., Favreau F., Faye P.-A., Vignoles P., Sturtz F., Trouillas P., Vallat J.-M. (2018). Local low dose curcumin treatment improves functional recovery and remyelination in a rat model of sciatic nerve crush through inhibition of oxidative stress. Neuropharmacology.

[76] Yang G., Chang C.-C., Yang Y., Yuan L., Xu L., Ho C.-T., Li S. (2018). Resveratrol Alleviates Rheumatoid Arthritis via Reducing ROS and Inflammation, Inhibiting MAPK Signaling Pathways, and Suppressing Angiogenesis. J. Agric. Food Chem..

[77] Song S., Regan B., Ereifej E.S., Chan E.R., Capadona J.R. (2022). Neuroinflammatory Gene Expression Analysis Reveals Pathways of Interest as Potential Targets to Improve the Recording Performance of Intracortical Microelectrodes. Cells.

[78] Bedell H.W., Schaub N.J., Capadona J.R., Ereifej E.S. (2020). Differential expression of genes involved in the acute innate immune response to intracortical microelectrodes. Acta Biomater..

[79] Whitsitt Q.A., Koo B., Celik M.E., Evans B.M., Weiland J.D., Purcell E.K. (2022). Spatial Transcriptomics as a Novel Approach to Redefine Electrical Stimulation Safety. Front. Neurosci..

[80] Thompson C.H., Saxena A., Heelan N., Salatino J., Purcell E.K. (2021). Spatiotemporal patterns of gene expression around implanted silicon electrode arrays. J. Neural. Eng..

[81] Joseph K., Kirsch M., Johnston M., Münkel C., Stieglitz T., Haas C.A., Hofmann U.G. (2021). Transcriptional characterization of the glial response due to chronic neural implantation of flexible microprobes. Biomaterials.

[82] Song S., Druschel L.N., Chan R., Capadona J.R. (2023). Differential Expression of Genes Involved in the Chronic Response to Intracortical Microelectrodes under Review. Acta Biomater..

