# Supplementary material for: Fabrication Methods and Chronic In Vivo Validation of Mechanically Adaptive Microfluidic Intracortical Devices
Source: Micromachines (Basel). 2023 May 9;14(5):1015. doi: 10.3390/mi14051015 (PMC10223487; doi:10.3390/mi14051015)
Supplement: Supplementary file 1 [file micromachines-14-01015-s001.zip › micromachines-2334128-supplementary.pdf]

Supplementary Materials

Representative Mechanical Properties of As-Cast and Pressed Materials

Figure S1 is a representative plot of the stress-strain curve of as-cast films compared to pressed films.

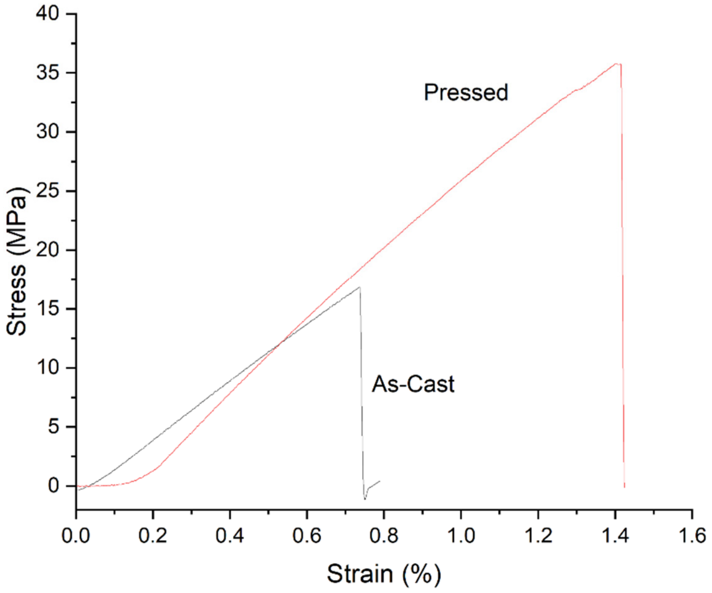

Figure S1. Representative stress-strain plot of as-cast and pressed films, corresponding to the conditions used to produce mold-only and mold-embossed microfluidic channels, respectively. Pressed films have a higher modulus, maximum stress, and maximum strain.

Cross-Sectional Analysis of Thermally Bonded Films

Figure S2 shows the cross-sections of thermally bonded films made using mold-only and mold-embossed methods. Both microfluidic channels are 100 µm wide.

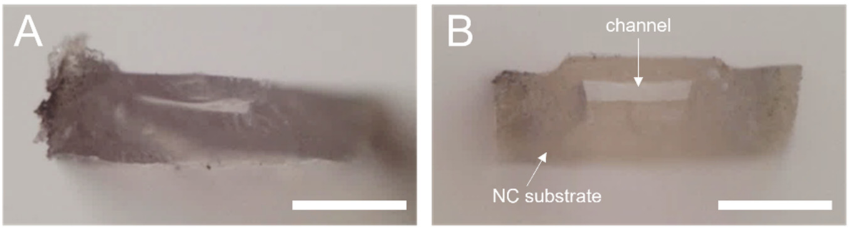

Figure S2. A) Cross-section of a 100 µm-wide channel microfluidic probe made using mold-only films. B) Cross-section of a 100 µm-wide channel microfluidic probe made using mold-embossed films.

Functionality Testing Modes of Failure

Figure S3 shows a functioning microfluidic probe. The probe, submerged in deionized water and connected to a syringe filled only with air and mounted on a syringe pump, should exhibit air bubbles eluting from the tip of the probe when the syringe is pushed.

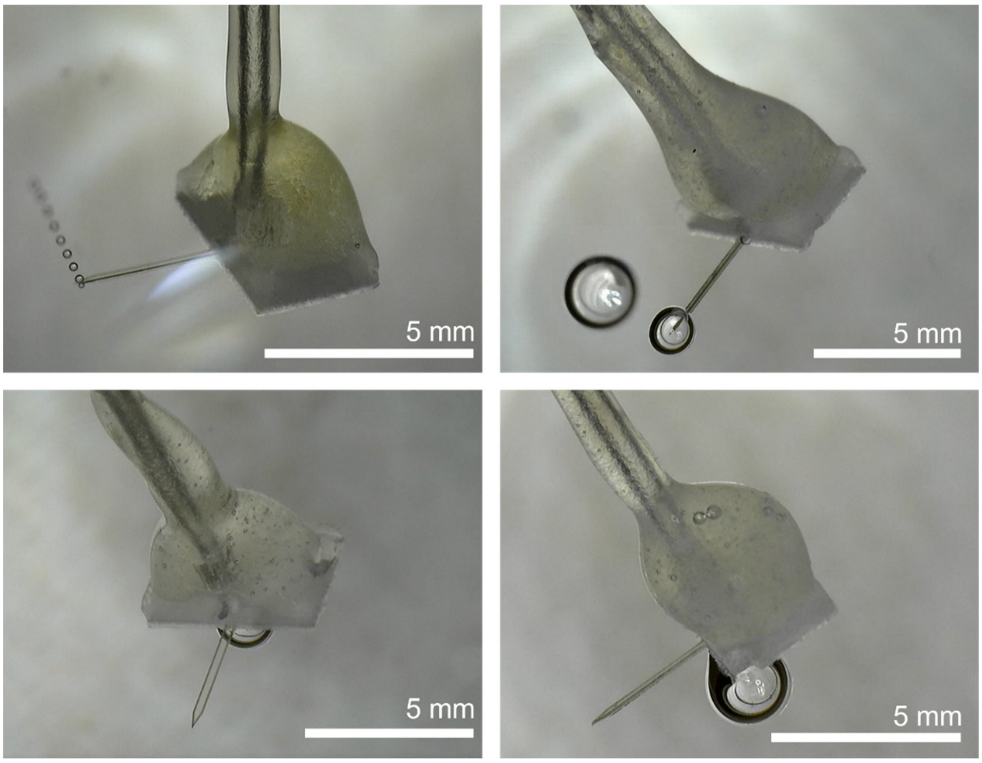

Figure S3. Top row) Successful probes showed clear air bubbles eluting from the tip of the probe when connected to a syringe pump flowing air. Bottom row) Failed probes with broken seals. The bubbles are not eluted from the tips of the probes, but instead at the interface of the connector and probe.

Functionality Testing Pressure Analysis

Table S1. The pressures exerted on the syringe pump during functionality testing. This table shows the force data for all 100 µm channel probes tested, regardless of success.

All Probes		
	Mold-Only (g)	Mold-Embossed (g)
Average	1800	191
Standard Deviation	2435	391
Max	8701	2416
Minimum	82	42

Initial In vivo implantation

Figure S4 shows an explanted probe with a 100 µm-wide microfluidic channel. Gross microscopy showed significant collapse of the channels in the center. Additionally, cellular debris was observed inside of the channels, which was further analyzed using confocal imaging of the DAPI stain (Figure S5) to observe cellular nuclei inside of the channels. Both channel collapse and occlusion by cellular debris may contribute to non-standard drug release rates.

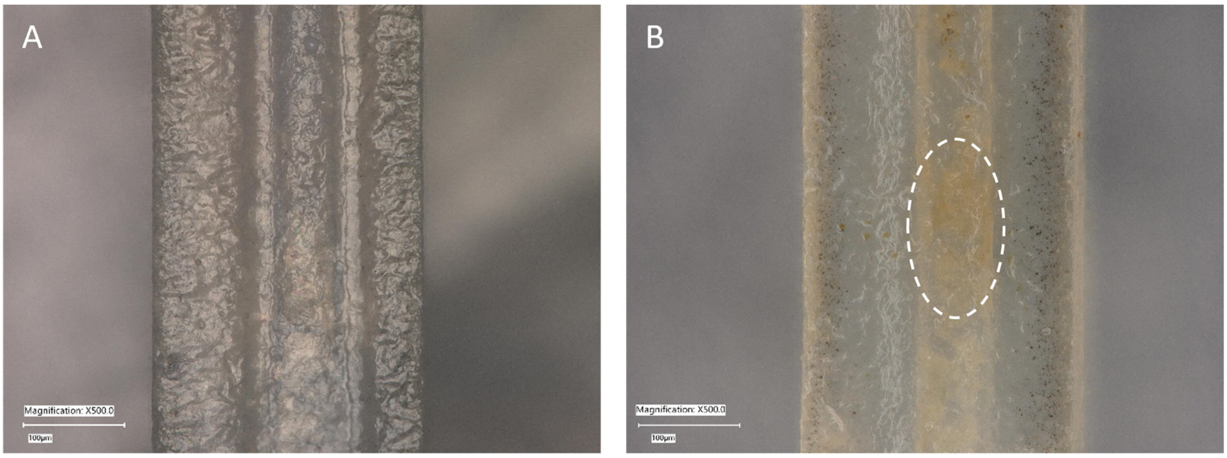

Figure S4. (A) Explanted probe image with collapsed channels. (B) Explanted probe with debris inside, confirmed to be cellular debris after DAPI staining and confocal imaging. Debris is outlined in the white dashed oval.

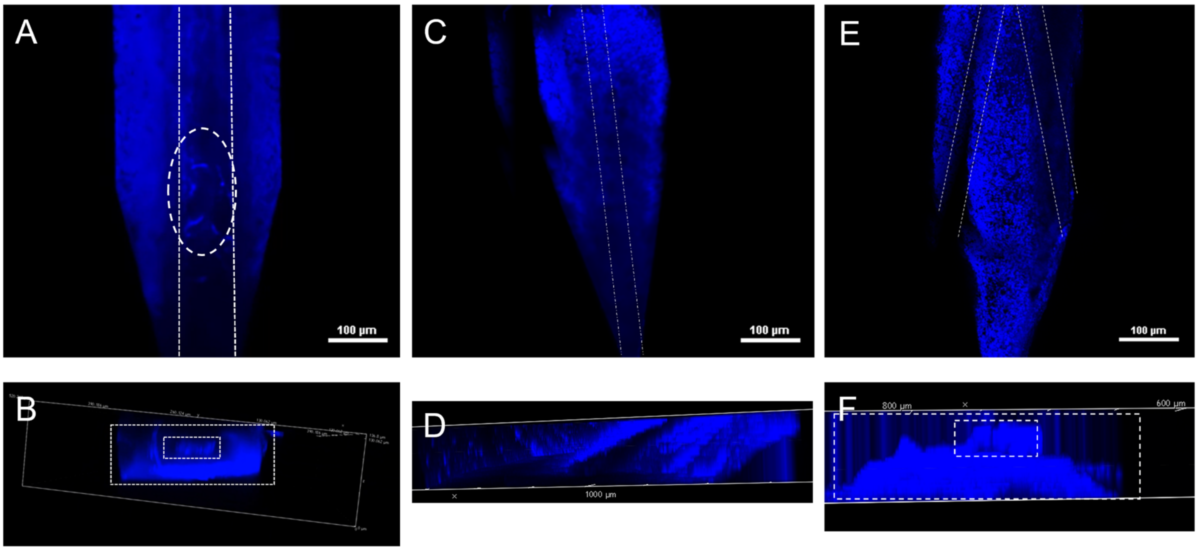

Figure S5. (A) Confocal 3D DAPI image of 100 µm wide channel explanted probes (S100-me). White outlines indicate the borders of the probe, showing cellular nuclei inside outlines with a white oval. (B) Confocal 3D DAPI image in cross-sectional view of 100 µm wide channel explanted probes (S100-me) with the inner white box indicating the channel inside which is filled with cellular nuclei. (C) Horizontal DAPI confocal image of 50 µm wide channel explanted probes (S50-me). (D) Cross-section view of image C. (E) Horizontal DAPI confocal image of 2 Outlet 50 µm wide channel probes (D50-me). F) Horizontal view of image E.

Second in vivo implantation with modified designs

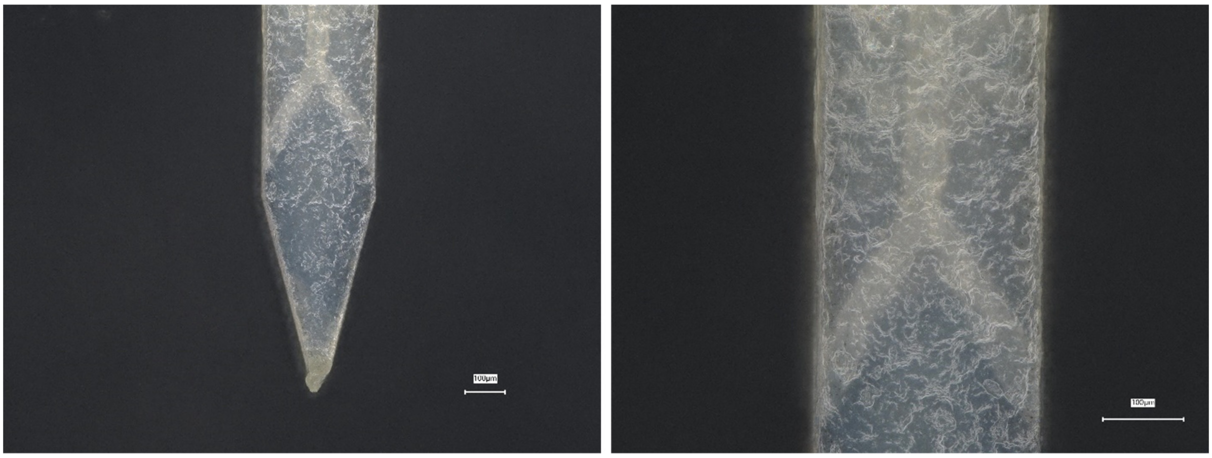

Figure S6. Gross microscopy images of explanted probes with altered branching design and 50 µm-wide channels.

Explanted devices in this pilot with the new designs and 50 µm-wide channels, showed that there was no collapse of the channels, and no debris inside of the channels as well. This was also reflected in the much more accurate flow rate of 0.15 ±0.03 µL/hr over the course of the whole study.
